# Case report: Herbal treatment of neutropenic enterocolitis after chemotherapy for breast cancer

**DOI:** 10.1515/biol-2022-0753

**Published:** 2023-10-24

**Authors:** Peng Xu, Chaoxiong Cui, Yukun Liu, Kun Fang, Qitang Wang, Chao Liu, Ruixia Tan

**Affiliations:** Galactophore Department, The Affiliated Qingdao Central Hospital of Qingdao University, The Second Affiliated Hospital of Medical College of Qingdao University, No. 127, Si-Liu South Road, Qingdao 266042, China; Ophthalmology Department, The Affiliated Qingdao Central Hospital of Qingdao University, The Second Affiliated Hospital of Medical College of Qingdao University, No. 127, Si-Liu South Road, Qingdao 266042, China; Health Management Department, Ophthalmology Department, The Affiliated Qingdao Central Hospital of Qingdao University, The Second Affiliated Hospital of Medical College of Qingdao University, No. 127, Si-Liu South Road, Qingdao 266042, China; Health Management Department, The Affiliated Qingdao Central Hospital of Qingdao University, The Second Affiliated Hospital of Medical College of Qingdao University, No. 127, Si-Liu South Road, Qingdao 266042, Shandong, China

**Keywords:** breast cancer, chemotherapy, neutropenic enterocolitis, Chinese herbal medicine

## Abstract

In this case report, a 53-year-old woman was diagnosed with severe NE after receiving chemotherapy for breast cancer. The patient with breast cancer was treated with a single cycle of docetaxel (140 mg) + epirubicin (130 mg) + cyclophosphamide (0.9 g) chemotherapy. However, the woman presented with symptoms of fatigue and diarrhea 5 days later accompanied with severe neutropenia according to the routine blood test. The computed tomography examination displayed the thickening and swelling of the colorectal wall. After the diagnosis of NE, the woman received antibiotics and supportive treatment, but her symptoms were not improved. The Chinese herbal medicine (CHM) diagnostic pattern was then designed for the patient. The patient was administered with two CHM decoctions. One decoction contained 24 kinds of herbal materials, and the other one was called pure ginseng decoction. These two decoctions were administered to the patient 2 or 3 times per day to tonify the spleen, nourish Qi and blood, and remove phlegm and damp heat symptoms. After the CHM treatment lasting for 10 days, the symptoms of the patient were improved, and she was discharged. In conclusion, CHM treatment played an indispensable role in curing the woman with chemotherapy-induced NE.

## 1 Introduction

Neutropenic enterocolitis (NE), also known as typhlitis, is a life-threatening inflammatory disorder of the intestine associated with neutropenia, which is characterized by an acute necrotizing process involving segments of the large and small intestines in the setting of agranulocytosis [1,2]. NE mainly occurs in immunosuppressed patients, and the mortality rate of NE is 30–50% [2].

NE is typically characterized by abdominal pain and fever in a neutropenic patient [3]. The entire intestine is usually affected, with the most impacted region occurring from the end of the ileum to the cecum and the ascending colon [4]. Laboratory test results include pancytopenia, hypoalbuminemia, and electrolyte imbalance such as hyponatremia, hypophosphatemia, and hypokalemia [1,5,6]. The diagnosis of NE depends on radiology. Computed tomography (CT) and ultrasonography are the best radiographic methods for NE early diagnosis at present [7,8]. Bowel wall thickening is regarded as a main diagnostic criterion, which can be determined through CT or ultrasonography [8]. The improvement of NE outcome relies upon the deep understanding of the risks for the condition, application of empiric therapy with broad-spectrum antimicrobial agents, systemic antifungal treatment, and meticulous attention to supportive care [9].

Natural killer (NK) cells, as important innate immune regulatory cells, play a key role in anticancer immunity and neutropenia [10,11,12]. The activation of NK cells is based on the balance of activating and inhibitory receptors expressed on the NK cell surface [10]. Upon activation, NK cells control the release of cytotoxic molecule and proinflammatory cytokines through immediate or short-lived immune response [12]. By transmitting signals from one cell type to another, cytokines are essential for communications between diverse cells in complex immune system responses [10]. The cytokines can eliminate target cells, for example, cancer cells, and activate other cells in the immune system such as T cells [12,13]. NK cells were previously reported to cooperate with neutrophils to damage the blood–brain barrier [14]. In the neoplastic process, NK cells and neutrophils have anti-tumor and pro-tumor functions because of high plasticity [15]. Neutrophils account for 40–70% of blood-nucleated cells in humans, and an increase in neutrophils in blood often is responsible for a sign of poor prognosis during cancer progression [15]. There exists a strong heterogeneity of the immune response between individuals, which may result from environmental and genetic factors, diet, lifestyle, or the interactions between these factors [16]. For example, vitamin D protects the innate immune system while inhibiting adaptive immunity, as evidenced by the stimulation of Th2 and Treg as well as the inhibition of Th1 and Th17, suggesting that diet is a variable factor affecting the function of the immune system [16]. Recently, many Chinese herbs or Chinese herb-derived compounds have been reported to affect immune cell populations in the immune (tumor) microenvironment. For example, solamargine was reported to alter dendritic cells, myeloid-derived suppressor cells, and T-cell populations by regulating macrophages and thus alleviate the immunosuppressive microenvironment in hepatocellular carcinoma [17]. The traditional Chinese medicine Yi-Yi-Fu-Zi-Bai-Jiang-San alters Tregs to hamper colorectal cancer cell growth [18].

In our case, a decoction containing 24 types of herbal materials and ginseng decoction were administered to a patient with breast cancer who was diagnosed with NE during chemotherapy. This case report revealed the protective role of Chinese herbal medicine (CHM) treatment against the alteration of neutrophils and white blood cell (WBC) counts in NE. Our report may provide more evidence supporting the function of CHM in immune system-related diseases such as NE.

## 2 Case presentation

A 53-year-old woman was admitted to the hospital with symptoms of diarrhea and fatigue. Five days before these symptoms, she had finished her first cycle of chemotherapy with docetaxel (140 mg) + epirubicin (130 mg) + cyclophosphamide (0.9 g) due to her left breast cancer (ycT2N1M0, stage IIB, Luminal B negative). She presented frequent diarrhea 2–3 times per day without the symptom of abdominal pain or distention, and the stools were loose and yellow.

**Figure 1:**
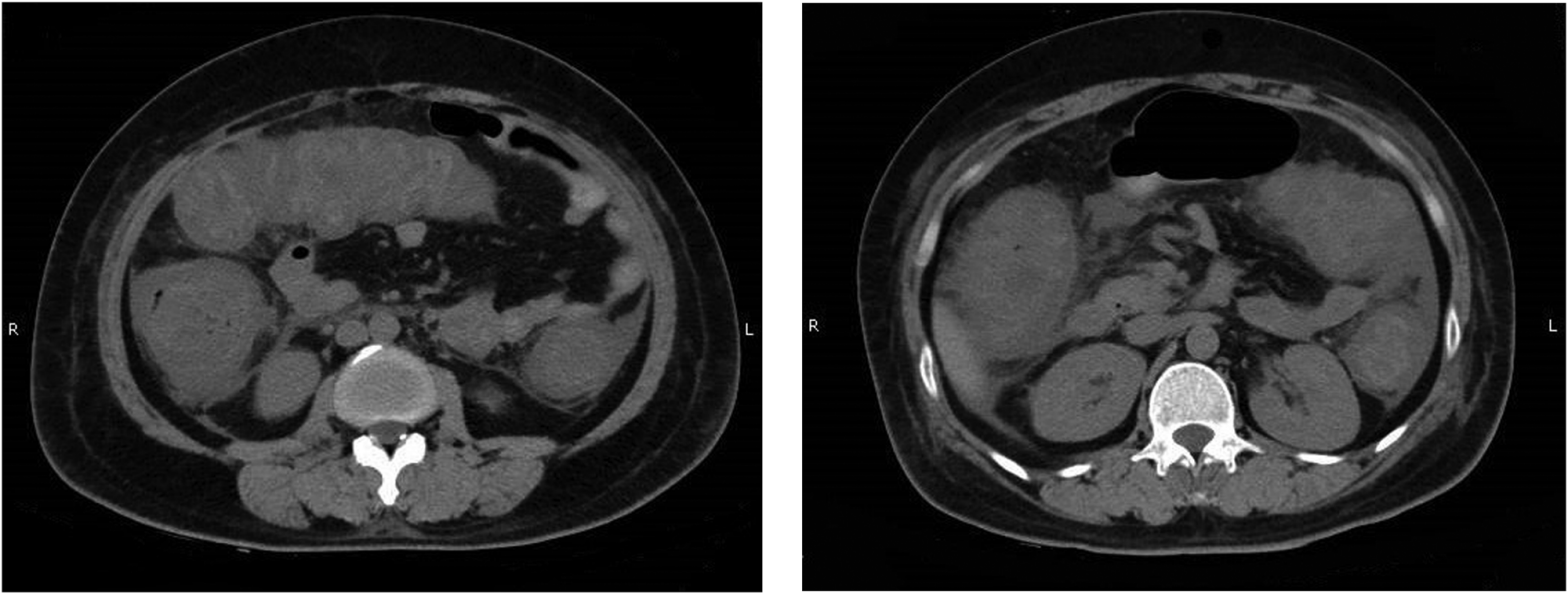
CT scan findings on August 7, 2022. These scan images showed thickening and swelling of the colorectal wall, abdominal and pelvic effusion, and a blurred abdominal fat space.

On July 29, her blood test showed severe myelosuppression. The WBC count was 0.65 × 10^9^/l, and the neutrophil count was 0.02 × 10^9^/l. The concentration of C-reactive protein (CRP) was 239.65 mg/l, and that of procalcitonin (PCT) was 0.32 ng/ml. Considering the severe myelosuppression after chemotherapy, the patient was primarily diagnosed with acute agranulocytosis. The recombinant human granulocyte colony-stimulating factor (200 μg, hypodermic injection, qd) was intramuscularly administered to the patient for 3 days to increase the number of WBC and neutrophils. Cefoperazone/sulbactam (3 g + 100 ml in 0.9% sodium chloride injection, intravenous injection, q12h) was intravenously administered to the patient for 6 days to prevent severe bacterial infection. On August 1, she complained of a fever of 40℃. The frequency of diarrhea increased to 5–6 times per day. Blood cultures for the diagnosis of bacterial and fungal infections were negative. There exist no abnormalities in the results of the stool examination, while the occult blood test was positive. The results of fecal flora analysis were as follows: (1) 70% of Gram-negative bacilli, (2) 20% of Gram-positive cocci, (3) 10% of Gram-positive bacilli, and (4) occasional Gram-stained fungi. We increased the use of antidiarrheals and administered Peifeikang (Shanghai Xinyi Pharmaceutical Co., Ltd.) to her. Peifeikang are probiotics that can replace the pathogenic flora in the intestine. The above-mentioned treatment was continued. On August 2, the results of blood tests were as follows: WBC count: 3.10 × 10^9^/l, neutrophil count: 1.90 × 10^9^/l, CRP concentration: 216.6 mg/l, and PCT concentration: 1.37 ng/ml. On August 4 and 5, the WBC count was 13.36 × 10^9^/l and 24.46 × 10^9^/l, and the neutrophil count was 11.06 × 10^9^/l and 21.96 × 10^9^/l, respectively. The number of WBC and neutrophils in her blood samples was increased rapidly, while the value of CRP suggested that there may exist a serious infection. Thus, we administered vancomycin (1.0 g + 250 ml in sodium chloride injection, iv, q12h) to her for the following 13 days. On August 7, the patient still suffered from fever, and the frequency of diarrhea increased to 6–12 times per day. The results of fecal floral analysis were as follows: (1) 90% of Gram-negative bacilli, (2) 10% of Gram-positive cocci, and (3) negative Gram-stained fungi. The results of blood tests showed that the number of WBC and neutrophils was 46.47 × 10^9^/l and 41.87 × 10^9^/l, respectively. Despite the use of antimicrobials, WBC and granulocyte counts were continuously elevated. Meanwhile, the symptoms of fever and diarrhea were not improved in the context of intestinal dysbiosis. A CT scan was then performed, and the scan image showed that the colorectal wall was thickened and swelled; there was effusion in abdomen and pelvis, and abdominal fat space was blur (Figure 1). After a multidisciplinary consultation with the CT report, she was diagnosed with NE.

After that, human albumin and plasma were infused to the patient for improvement of coagulation, osmotic pressure, and hemodynamics. Additional tests for pathogen detection were negative. On August 24, the blood parameters were not significantly improved. The fecal flora analysis indicated that the intestinal flora was significantly reduced, while bacterial cultures of specimens obtained from blood, pleura, and peritoneum were negative. Further application of antibacterial drugs may further aggravate intestinal dysbiosis, which is unbeneficial for recovery. Subsequently, we decided to use CHM for the following treatment. After our consultation with the CHM department, her diagnostic pattern was considered “phlegm-dampness due to spleen and stomach deficiency as well as asthenia of Qi and blood.” A therapy should be designed to tonify the spleen, nourish Qi and blood, and remove phlegm and damp heat.

**Figure 2:**
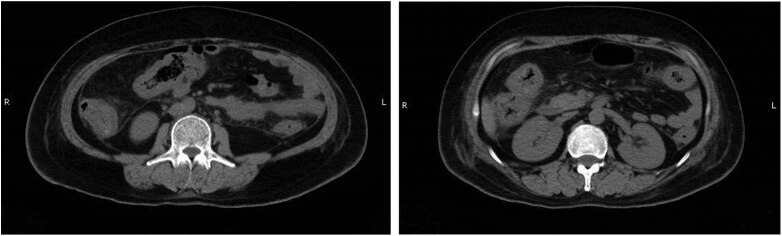
CT scan images on September 7, 2022. After CHM treatment, the cecum and descending colon were normal, while there was a small effusion of blood in the abdominal cavity. There is an edematous thickening of the ascending and transverse colon.

**Figure 3:**
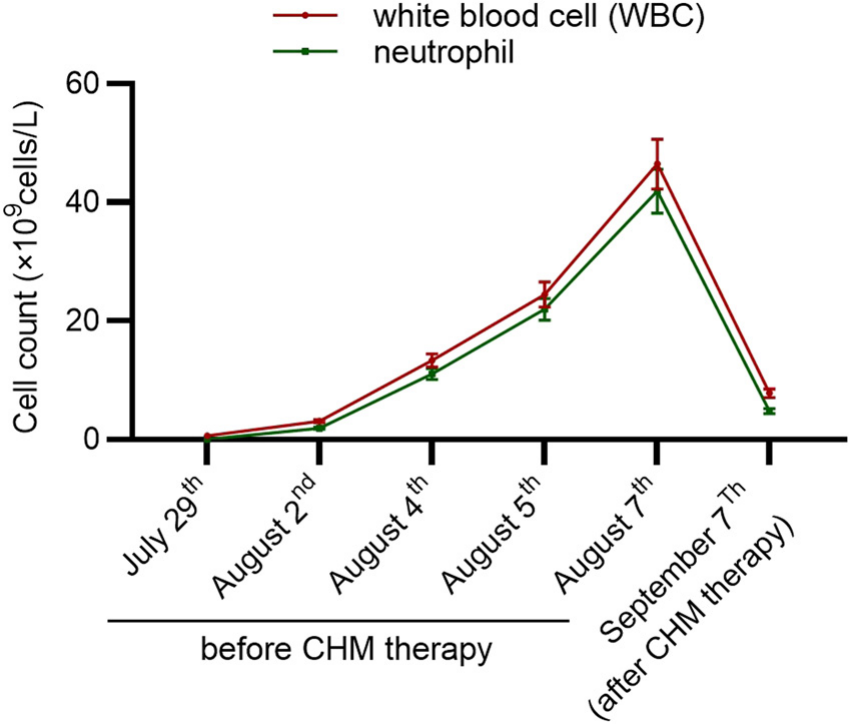
Changes in WBC and neutrophil counts before and after CHM treatment. A line graph was drawn to illustrate the changes of cell counts before and after therapy. Severe myelosuppression was found on July 29. From August 2 to August 7, the cell counts increased rapidly and reached the peak on August 7; however, the symptoms of fever and diarrhea were not improved. After CHM treatment, the cell counts recovered on September 7.

Therefore, a traditional Chinese medicine decoction including 24 types of Chinese medicinal herbs was prepared. The names and gram weight of these Chinese medicinal herbs are summarized in Table 1. These herbal materials were prepared in three doses, and each dose was decocted and administered to the patient 2 or 3 times per day. A pure ginseng decoction, with ginseng being decocted with red sugar and water, was also administered to her. Three days after CHM treatment and enteral nutrition, the frequency of diarrhea was reduced from 12 times to 5–6 times per day. The symptoms of fever, chills, and stool consistency were ameliorated, and the patient was more energetic than before. The decoctions were administered to her until recovery. On September 7, the results of blood tests showed that WBC and neutrophil counts were 7.81 × 10^9^/l and 4.76 × 10^9^/l, respectively. The results of the CT examination showed an edematous thickening of the ascending and transverse colon. The cecum and descending colon were normal, while there was a small effusion of blood in the abdominal cavity (Figure 2). The changes in WBC and neutrophil counts before and after CHM treatment are shown in Figure 3. On September 8, 10 days after the CHM treatment, the patient was in good condition and she was discharged from our hospital.

**Table 1:** The Chinese medicinal herbs used in this study

Name of Chinese medicinal herbs	Gram weight
**Decoction 1 (24 herbal materials)**	
Codonopsis pilosula	30 g
Poria cocos	20 g
Atractylodes macrocephala	12 g
Amomum villosum	10 g
Cyperus tuberose	12 g
Ginger Pinellia ternata	15 g
Pericarpium citri	12 g
Bamboo shavings	15 g
Astragalus membranaceus	50 g
Radix Rehmanniae	30 g
Ginseng psammophila	30 g
Ophiopogon japonicus	20 g
Trichosanthin	30 g
Dendrobium Nobile	15 g
Honeysuckle	20 g
Fritillaria thunbergii	10 g after mixing with water
Fructus Tritici Levis	30 g
Ephedra root	15 g
Coix Jobi	30 g
Chinese yam	30 g
Gorgon fruit	30 g
Semen dolichoris album	30 g
Fried hawthorn	20 g
Fried malt	20 g
**Decoction 2**	
Pure ginseng	

On October 13, 35 days after the discharge, the patient’s blood routine as well as liver and kidney functions were basically normal. Then, the patient underwent modified radical surgery for left breast cancer under general anesthesia. Afterwards, the patient underwent an EC sequential T chemotherapy regimen after the surgery. By the time of finishing this article, the patient had received 4 cycles of epirubicin (150 mg), cyclophosphamide (1 g), and docetaxel (400 mg).


**Informed consent:** Informed consent has been obtained from all individuals included in this study.
**Ethical approval:** The research related to human use has been complied with all the relevant national regulations, institutional policies, and in accordance with the tenets of the Helsinki Declaration and has been approved by the Institutional Ethics Committee of Affiliated Qingdao Central Hospital of Qingdao University, Qingdao Cancer Hospital (protocol code [Y]KY202305701 and date of approval: March 30, 2023).

## 3 Discussion

NE is a life-threatening digestive complication often reported in children with leukemia and is rare in adults [3,19]. A few articles reported that adult patients with breast cancer were diagnosed with NE after receiving chemotherapy [20,21]. For example, a 22-year-old woman developed NE despite the use of granulocyte colony-stimulating factor after chemotherapy for breast cancer, and the patient underwent surgery for severe abdominal pain [20]. After receiving neoadjuvant chemotherapy for epidermal growth factor receptor 2 positive left breast cancer, a 48-year-old woman was found to have neutropenia and was treated for neutropenic sepsis in the emergency department [21]. Cherri et al. concluded that taxanes seem to be the most involved chemotherapy drugs for the induction of NE in solid tumor treatment, although the exact incidence is unknown [20]. In this case report, the patient presented a severe lack of granulocytes in the blood test and had frequent diarrhea with a high fever. Moreover, the CT scan images displayed the thickening and swelling of the colorectal wall, abdominal and pelvic effusion, and a blurred abdominal fat space, and therefore, the diagnosis of NE was confirmed. Currently, NE is diagnosed when the thickness of the intestinal wall equals to or more than 4 mm at the onset of at least one of the following symptoms: (1) axillary temperature ≥38°C, (2) abdominal pain, and (3) diarrhea defined as more than three liquid stools per 24 h in the presence of neutropenia (absolute neutrophils count <0.5 × 10^9^/l) [4].

There is no unified treatment standard for NE at present and the pathogenesis is not fully understood [22]. Early recognition, appropriate risk-stratified management, and symptomatic and supportive treatment can prevent the application of surgery and unnecessary antibiotics and can avoid complications and mortality [23]. The duration of antibiotic therapy for NE is controversial [4], and surgery has a high risk of bleeding, postsurgical infections, and damaged wound healing, which only suits the NE patients with complications [23]. The most common microbiologically proven complication of NE is bloodstream infection (BSI) can be induced by bacteria such as Escherichia coli [24]. Prompt administration of broad-spectrum empiric antibiotics is critical to prevent progressive disease, severe sepsis, and even death [25,26]. The incidence of BSI in adults is higher than in children, which is the leading cause of death [27]. To prevent BSI and sepsis, the patient in our study was given cefoperazone sulbactam for 6 days and vancomycin for the next 13 days. Although the blood culture showed negative results, the imbalance of intestinal flora was further aggravated, and her symptoms were not improved at all. The clinicians must balance the relationship between the risk of follow-up NE treatment (e.g., antibiotic resistance and metronidazole-neurotoxicity) and the patient’s total benefits. Shane reported that the rapid recovery of neutrophils usually turned into a good outcome of NE [4]. Unfortunately, in the present study, even though the WBC and neutrophil counts in blood samples of the patient reached the panic value of 46.47 × 10^9^/l and 41.87 × 10^9^/l, the patient’s symptoms of fever and diarrhea were still out of remission. Finally, human albumins were given to improve blood coagulation, osmotic pressure, and hemodynamics. However, the unsatisfied outcome and long-term treatment highly consumed the patient’s and attending physicians’ patience.

The balance of intestinal flora plays an essential role in human immunity [7]. NE seems to start with intestinal mucosal injury together with neutropenia and the weakened immune system of the afflicted patients [20,21,28]. The factors leading to NE are mucosal injury, cecal distension with resultant ischemia, cytotoxic drugs, and microbiological agents. Blocking these injuries would be the key to NE treatment and rehabilitation of intestine.

The improvement of her symptoms was brought about by the tentative use of CHM. Previous studies validate that the adjunctive use of CHM with chemotherapy may reduce the adverse events induced by chemotherapeutic agents, including nausea and vomiting, diarrhea, alopecia, myelosuppression, and impaired immune function [29]. Different natural products derived from CHM were identified with various anti-cancer properties, such as anti-proliferative, pro-apoptotic, anti-metastatic, and anti-angiogenic effects; the natural compounds can also regulate autophagy, reverse multidrug resistance, maintain the immunity balance, and improve the efficiency of chemotherapy [30,31,32]. Many studies found that CHM could effectively alleviate adverse gastrointestinal reactions (including diarrhea, nausea, and vomiting) to this anti-cancer chemotherapy-related therapy and decrease the incidence of bone marrow suppression [33]. Our case suggested a way to treat NE after chemotherapy with CHM. Moreover, some herbal materials mentioned in our medicine decoction have previously been investigated and the mechanisms underlying their spleen-invigorating function and regulatory role in gut microbiota are partly explained. For example, Codonopsis pilosula polysaccharide contained in Codonopsis Radix was reported to improve spleen deficiency by attenuating gut microbial dysbiosis [34]. Pori cocos oligosaccharides, a component in Pori cocos, have been revealed to alleviate dextran sodium sulfate (DSS)-induced colitis by modulating gut microbiota dysbiosis [35]. A new polysaccharide named CYP-1 isolated from Chinese yam has a therapeutic effect on gut microbiota dysbiosis by reducing the abundances of *Alistipes*, *Helicobacter*, and an unidentified Enterobacteriaceae in DSS-induced colitis [36]. CHMs also exert therapeutic impacts on other bacterium infections. For example, Ginseng treatment can reduce bacteria including Deferribacters, *Lactobacillus*, and *Helicobacter* against various diseases [37]. CHM is effective against the infection of human respiratory syncytial virus by stimulating IFN-β secretion [38]. Therefore, it can be concluded that the auxiliary therapeutic effect of CHM on NE is beyond doubt and of great potential.

## 4 Conclusions

This case report may strengthen the existing knowledge about CHM treatments for NE, especially for the refractory cases with poor therapeutic effects, and CHM may be another option after all efforts.
